# Stability and gene strand bias of lambda prophages and chromosome organization in *Escherichia coli*

**DOI:** 10.1128/mbio.02078-23

**Published:** 2024-06-18

**Authors:** Xintian Li, Oscar Gallardo, Elias August, Bareket Dassa, Donald L. Court, Joel Stavans, Rinat Arbel-Goren

**Affiliations:** 1 Gene Regulation and Chromosome Biology Laboratory, National Cancer Institute, Frederick, Maryland, USA; 2 Department of Physics of Complex Systems, Weizmann Institute of Science, Rehovot, Israel; 3 Department of Engineering, Reykjavik University, Reykjavík, Iceland; 4 Department of Life Sciences Core Facilities, Weizmann Institute of Science, Rehovot, Israel; University of Pittsburgh, Pittsburgh, Pennsylvania, USA; University of Maryland, College Park, Maryland, USA

**Keywords:** gene strand bias, lysogen stability, horizontal gene transfer, evolution, bacteria, temperate bacteriophage, chromosome organization

## Abstract

Temperate phage-mediated horizontal gene transfer is a potent driver of genetic diversity in the evolution of bacteria. Most lambdoid prophages in *Escherichia coli* are integrated into the chromosome with the same orientation with respect to the direction of chromosomal replication, and their location on the chromosome is far from homogeneous. To better understand these features, we studied the interplay between lysogenic and lytic states of phage lambda in both native and inverted integration orientations at the wild-type integration site as well as at other sites on the bacterial chromosome. Measurements of free phage released by spontaneous induction showed that the stability of lysogenic states is affected by location and orientation along the chromosome, with stronger effects near the origin of replication. Competition experiments and range expansions between lysogenic strains with opposite orientations and insertion loci indicated that there are no major differences in growth. Moreover, measurements of the level of transcriptional bursts of the *cI* gene coding for the lambda phage repressor using single-molecule fluorescence *in situ* hybridization resulted in similar levels of transcription for both orientations and prophage location. We postulate that the preference for a given orientation and location is a result of a balance between the maintenance of lysogeny and the ability to lyse.

**IMPORTANCE:**

The integration of genetic material of temperate bacterial viruses (phages) into the chromosomes of bacteria is a potent evolutionary force, allowing bacteria to acquire in one stroke new traits and restructure the information in their chromosomes. Puzzlingly, this genetic material is preferentially integrated in a particular orientation and at non-random sites on the bacterial chromosome. The work described here reveals that the interplay between the maintenance of the stability of the integrated phage, its ability to excise, and its localization along the chromosome plays a key role in setting chromosomal organization in *Escherichia coli*.

## INTRODUCTION

Comparative phylogenetic analyses have demonstrated that microbial genome evolution can be described by periods of gradual gene loss, punctuated by episodes of gene gain through horizontal gene transfer, with the latter providing the principal route of evolutionary innovation in bacteria (1, 2). These studies have further demonstrated that lysogenization of temperate bacteriophages is the most prevalent form of horizontal gene transfer. Prophages are found in almost all sequenced pathogenic and non-pathogenic bacterial genomes, where they contribute by lysogenic conversion to bacterial fitness (3, 4). In *Escherichia coli*, the number of prophages can range between 2 and 20, depending on the strain (5). For instance, prophage sequences constitute 13.5% of the genome of O157:H7 strain EC4115. Phages, once integrated, can either excise their genome and enter the lytic pathway, as exemplified by bacteriophage lambda (6), or they may lose the ability to excise, in which case they adapt to their host genome, conferring traits that help bacterial cells cope with adverse environmental conditions (7). It has been shown that prophages are not randomly distributed among the four macrodomains of the *E. coli* chromosome, and within the macrodomains, they are more abundant in regions closer to the terminus of replication (8). The choice of integration sites is suggested to occur in ways that minimize detrimental effects on chromosomal organization and cell survival (8–10).

Intriguingly, prophages and other horizontally transferred genes such as integrative conjugating elements display frequent co-orientation when integrated in bacterial chromosomes (8, 11–13). In *E. coli*, lambdoids represent about 50% of prophages (8). It has been noted that natural lambdoid prophages are mostly found integrated in the same orientation relative to the direction of propagation of the replication fork (11). This is neither limited to lambdoid phages nor Gram-negative bacteria (14), and a mechanism involving the collisions of DNA and RNA polymerases has been suggested to be the main cause for this marked asymmetry (8). In fact, this mechanism was originally proposed to explain gene strand bias of essential genes in bacteria (15, 16). The typical speeds of DNA and RNA polymerases differ by a factor of about 10, so collisions are unavoidable, both in the leading and lagging strands. The effects are expected to be larger when a gene is coded in the lagging strand since the DNA and RNA polymerases collide head-on. In contrast, collisions are minimized when transcription is on the leading strand, where replication is continuous. For about 90% of lambdoid prophages, transcription of their repressor gene is oriented against replication, suggesting that replication-transcription collisions are not responsible for the observed orientation bias. Indeed, the inversion of lambda orientation at different sites was reported to create no measurable growth defects or genetic instabilities, and it was hypothesized that small selective effects iterated over many generations could account for the observed pattern of integration orientation (11). However, no evidence in support of this hypothesis has been provided. It has been pointed out that the elevated rates of adaptive mutations in lagging-strand genes are less than compelling as an explanation for gene orientation preference (17). Here, we revisited the issue of the preference of lambdoid prophages to co-orient with the replication fork in *E. coli* by comparing the stability of prophages in the lysogenic state at the same site but integrated in opposite directions. This directional effect has been tested at different locations on the chromosome.

The stability of the lysogenic state is maintained by the lambda CI repressor, which stimulates continued transcription of the *cI* gene. CI forms an octamer that is needed for cooperative, effective repression of the P_RM_ promoter to fine-tune the rate of switching from lysogeny toward the lytic pathway (18, 19). We assessed prophage stability in two ways. We measured the amount of free phage in solution, as produced by SOS-dependent spontaneous and non-spontaneous induction in exponentially growing cultures of these lysogens (20–22), and we directly quantified transcription bursts from the *cI* gene, coding for the lambda repressor (21). In the latter case, statistics were obtained by measuring the number of cI mRNA molecules in individual cells, using single-molecule fluorescence *in situ* hybridization methods (smFISH) (23–26). Furthermore, we carried out bulk competition assays as well as range expansions, which are sensitive to small fitness advantages, to determine whether inversion of the integrated prophage leads to a cell growth disadvantage (27–30). Our study aimed at elucidating the role that prophage orientation and prophage location play in shaping the architecture of the bacterial chromosome.

## RESULTS

### Effects of inversion of the lambda prophage at its wild-type location on the bacterial chromosome

**Fig 1:**
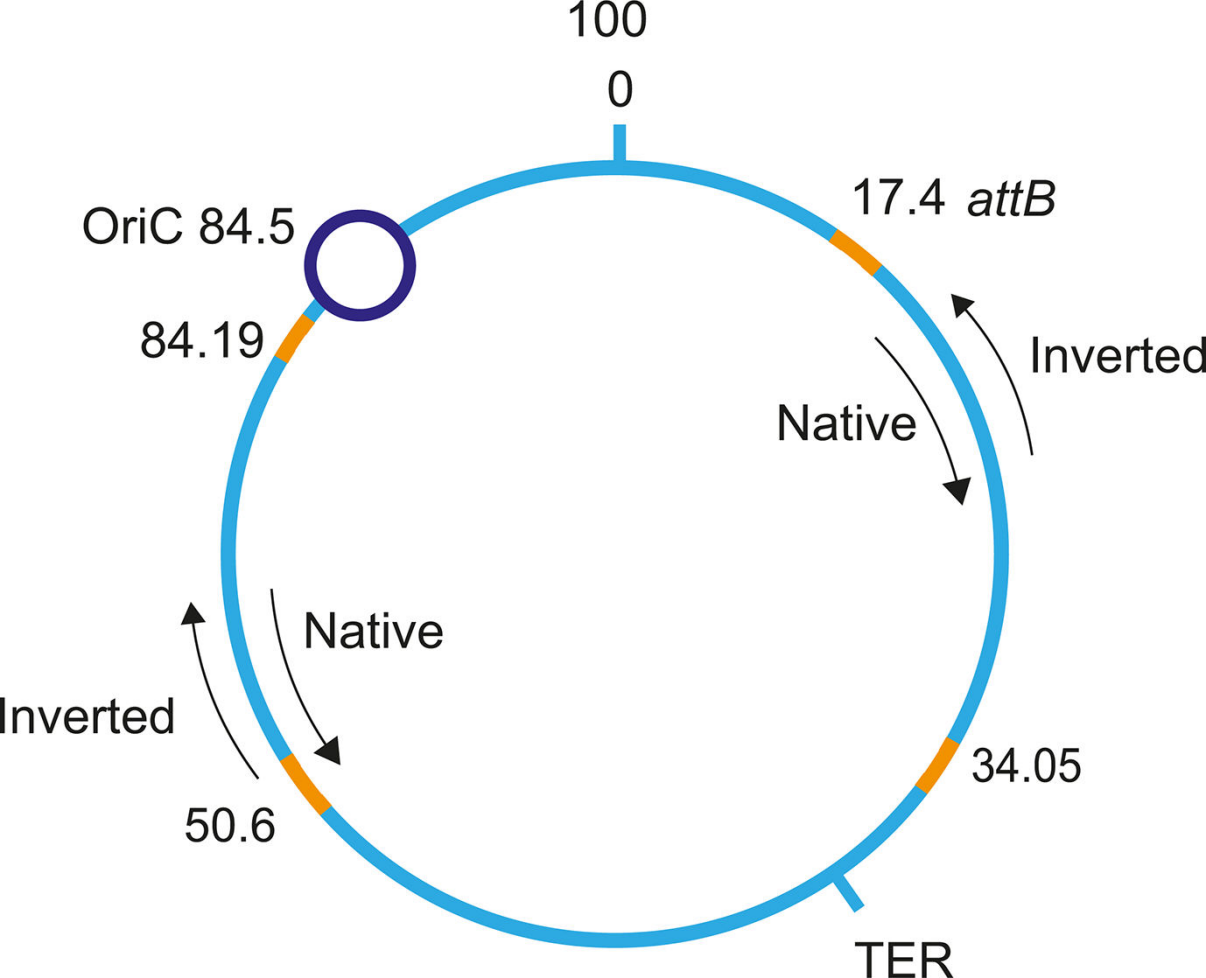
Lambda prophage insertion at four different *E. coli* genomic locations. Wild-type *attB* site (17.4′), a symmetric site on the other arm of the chromosome (50.6′), near the origin of replication (OriC, at 84.19′), and near the terminus of replication (TER, 34.05′). Lysogens with prophages integrated in either the native or in the inverted orientation were studied at 17.4′ and 50.6′. The bacterial chromosome is shown in cyan, and the prophages are in orange. The arrows represent the prophage integration orientations relative to the replication direction.

Prophage lambda stability was determined by comparing two prophage strains: one in which a lambda prophage is integrated in its native orientation, versus one in which the lambda prophage is in an inverted orientation. Using these two strains in exponentially growing cultures, we measured the number of free phages in solution produced by spontaneous induction of their respective lysogens, each of which bear an integrated prophage at the same site, but inverted relative to each other. We tested inverted prophages at the wild-type (WT) *attB* site, as well as at other locations along the bacterial chromosome as shown in Fig. 1. No significant change was found when the prophage was inverted at the wild-type *attB* site or other locations along the bacterial chromosome as shown in Table 1.

**TABLE 1:** Amount of free phage in solution produced by spontaneous induction in exponentially growing cultures of lysogens^*a*^

Strain	Position	Orientation	Phage release untreated	Ratio inv/nat untreated	Phage release mitomycin C	Ratio inv/nat treated
XTL856	17.4′	Native	(1.2 ± 0.36) × 10^6^	1.00 ± 0.37	(7.8 ± 3.6) × 10^10^	0.83 ± 0.42
XTL855	17.4′	Inverted	(1.2 ± 0.27) × 10^6^		(6.5 ± 1.4) × 10^10^	
XTL906	34.05′	Native	(3.5 ± 0.66) × 10^5^	0.60 ± 0.14	(2.2 ± 0.9) × 10^10^	0.50 ± 0.13
XTL905	34.05′	Inverted	(2.1 ± 0.25) × 10^5^		(1.1 ± 0.27) × 10^10^	
XTL892	50.6′	Native	(2.0 ± 0.11) × 10^5^	1.35 ± 0.13	(1.5 ± 0.04) × 10^10^	1.07 ± 0.03
XTL891	50.6′	Inverted	(2.7 ± 0.14) × 10^5^		(1.6 ± 0.03) × 10^10^	
XTL894	84.19′	Native	(5.7 ± 0.23) × 10^4^*	0.05 ± 0.02*	(8.6 ± 1.6) × 10^9^*	0.005 ± 0.002*
XTL893	84.19′	Inverted	(2.6 ± 0.92) × 10^3^*		(4.6 ± 0.95) × 10^7^*	

^*a*^ Bacterial strains were either untreated or induced by mitomycin C (1 mg/mL). The ratios between the inverted and native orientations at the four chromosome loci are shown both for untreated and treated cultures. The mean and standard error were determined from three independent repeats. Asterisks indicate statistical significance calculated by Mann–Whitney *U*-test (*P* < 0.05) between the two orientations at the same position. The values are normalized to 1 × 10^8^ cells.

**Fig 2:**
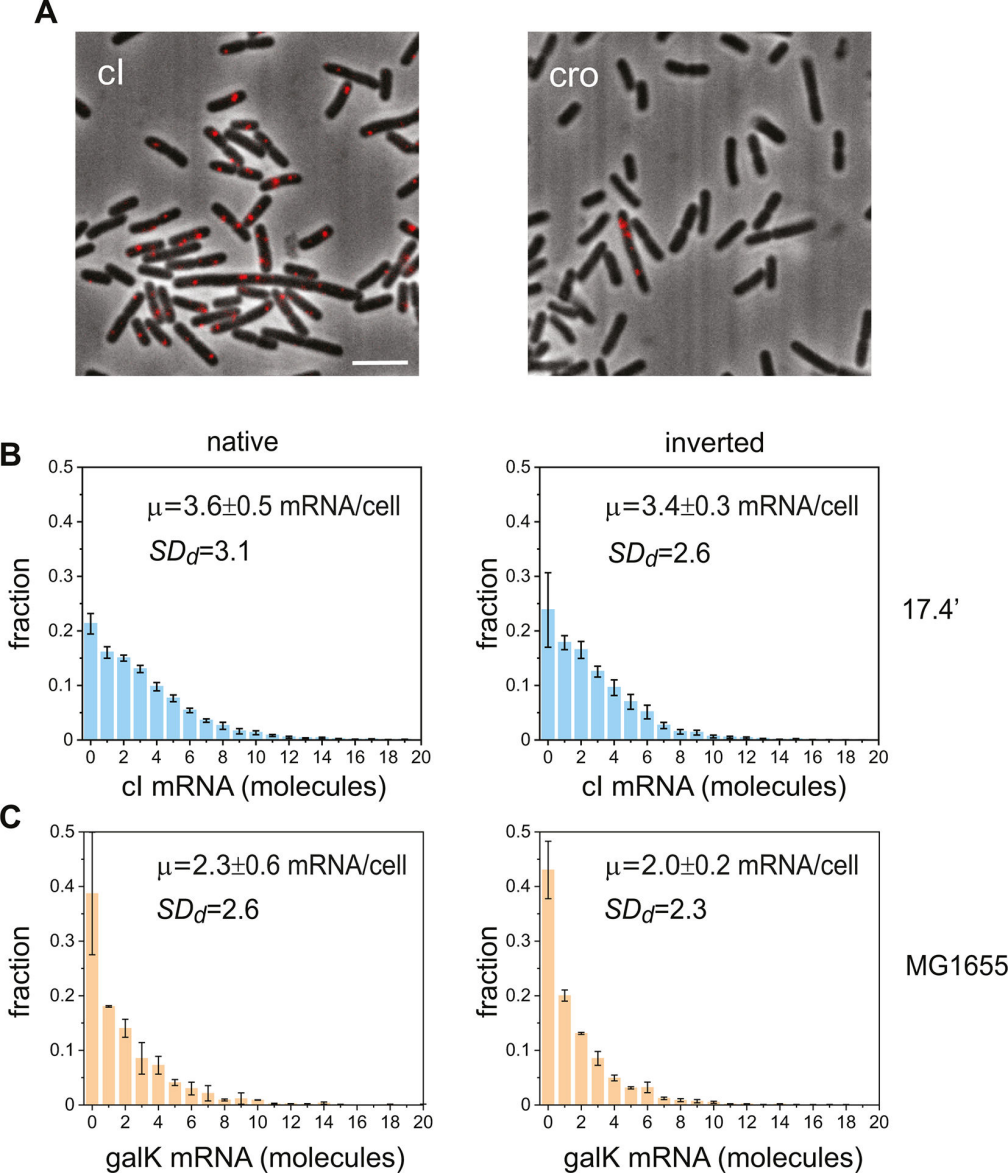
Transcript variability in lysogen cells bearing lambda prophages integrated in opposite orientations at the wild-type *attB* site 17.4′. (**A**) Typical snapshot of cI transcripts produced from the P_RM_ promoter in individual *E. coli* cells, visualized by smFISH (left). Snapshot of a cell expressing cro transcripts produced by spontaneous induction (right). The snapshots are overlays of phase contrast and fluorescence images, obtained from lysogens of lambda phage integrated in a native direction. The scale bar represents 5 µm. (**B**) Distributions of cI transcripts measured in lysogens integrated with native (left, XTL856) and inverted (right, XTL855) orientations. (**C**) Distributions of gal transcripts measured in the native (left) and inverted (right) orientations of the *galETK* operon in MG1655 background. The mean transcript number per cell, µ, as well as the standard deviation of the histograms averaged over independent experimental runs, ${SD}_{d}$, are given in each case for both panels B and C (Materials and Methods). All distributions represent an average of at least three independent experimental repeats (about 3,000 cells for each orientation), while error bars represent standard errors.

To further test for change in prophage stability upon inversion, we quantified the stochastic bursts in the transcription of the *cl* gene coding for the lambda repressor for both native and inverted prophage orientations at the lambda *attB* site. To this end, we carried out smFISH measurements of *cI* transcripts for the two orientations of the prophage, native and inverted. In Fig. 2A, we have shown a typical snapshot of *cI* transcripts in individual cells, obtained with native integration orientation and the histograms of transcript numbers per cell (Fig. 2B). The histograms corresponding to both orientations are similar, as quantified by the mean transcript numbers, the standard deviation of mRNA number $\left({SD}_{d}\right)$: the mean transcript numbers are 3.6 ± 0.5 mRNA/cell for the native orientation and 3.4 ± 0.3 mRNA/cell for the inverted one.

Finally, we estimated prophage stability by measuring the percentage of cells that spontaneously take the lytic pathway in each integration orientation by determining the number of cells that express lytic cro transcripts (Fig. 2A). We found that the number of cells with one or more cro transcripts represented 0.28% ± 0.09% of the total (about 3,500 cells) in the native orientation and 0.29% ±0.06% in the inverted orientation. This number is consistent with previously reported numbers of free phage produced by spontaneous induction (20).

### Effects of inverting the bacterial *galETK* operon

To test the effects of orientation inversion of genes in the *E. coli* chromosome, we compared the transcript levels of the *galK* gene coding for galactokinase as transcribed in the wild-type *galETK* operon, with the transcript levels obtained when the entire *galETK* operon is inverted. Note that for the wild type, the direction of transcription of the *gal* operon is opposite to the direction of chromosome replication. Histograms representing both orientations (Fig. 2C) are similar, as quantified by their value of ${SD}_{d}$. A Mann-Whitney *U*-test supports the hypothesis that the sample medians are the same in both orientations at the 0.05 confidence level.

### smFISH measurements of galK transcripts in lysogen backgrounds

To ascertain that the statistics of stochastic transcription events on a nearby bacterial gene are independent of the orientation of integration of lambda phage DNA at the wild-type *attB* site, we measured transcript number distributions from the *galK* gene in both native and inverted lysogens using smFISH. In Fig. S1A, we present the respective histograms. A Mann-Whitney *U*-test for the equality of population medians of the two histograms supports the hypothesis that the medians are the same in both cases at the 0.05 confidence level.

### Prophage stability between native and inverted orientations in a *recA^−^* background

We next checked whether the activation of the SOS network, which turns on the lambda lytic cycle through RecA-mediated degradation of CI, plays a role in prophage stability. In a *recA^−^* background, we measured cI transcript levels using smFISH in both prophage integration orientations. The observed transcript number distributions are comparable, with similar means and histogram standard deviations $SD_{d}$ (Fig. S1B). Our measurements of phage release of the native and inverted orientations (12 and 4.7 phages per 1 × 10^8^ cells, respectively) are significantly smaller than those obtained for the wild-type background strains, in line with previous observations (20). When these strains were treated with mitomycin C, we obtained 88 and 26 phages per 1 × 10^8^ cells, respectively. Sequencing of the *recA*^−^ strains showed a threefold higher number of mutations in the prophage sequence integrated in the native orientation relative to the other sequenced strains in this study (Table S3). In this strain, the cI sequence showed 98.17% identity with the cI857 sequence, while all the other sequenced strains were identical. These differences may be a result of both inversion of orientation and/or sequence alterations.

### Prophage stability at other integration sites along the *E. coli* chromosome

To shed light on the mechanisms behind the non-uniform distribution of lambdoid prophages along the *E. coli* chromosome as well as their biased integration orientation (8), we studied the stability of lambda prophages that were integrated at three locations other than the wild-type *attB* position. We studied a site located on the opposite arm of the bacterial chromosome at 50.6′, another site near the origin of replication 84.19′, and finally, a site near the terminus 34.05′ (Fig. 1). When the *attB* integration site was moved to a symmetric position on the opposite arm of the chromosome 50.6′, the ratio for the number of phages released in each prophage orientation was similar for the two positions on the chromosome (Table 1). However, absolute numbers of free phage released into solution by spontaneous induction, for prophage at the symmetric site at 50.6′, were about sixfold smaller than when integration was at the wild-type *attB* site at 17.4′ (Table 1). In contrast to the results observed at the wild-type *attB* site and its symmetric counterpart at 50.6′, the lambda prophage located near the origin of replication at 84.19′ has new features. First, there was more than an order of magnitude difference in the number of free phages released for the two prophage orientations (Table 1). Second, the number of free phages released at 84.19′ was more than two orders of magnitude smaller than for the wild-type *attB* site (Table 1).

The addition of mitomycin C and induction of the SOS response in all bacterial strains bearing prophages at 17.4′, 34.05′, and 50.6′ yielded more than four orders of magnitude higher phage release than for the untreated strains, without any significant effect of orientation (see Table 1). However, a lysogen whose attachment site was located near the origin of replication yielded about one order of magnitude smaller numbers of phage in the native orientation and two orders of magnitude less in the inverted orientation. While the trends are consistent with those observed in strains untreated with mitomycin C, the effects are quantitatively larger (Table 1).

The differences in orders of magnitude in phage release between the different integration locations of lambda prophages (Table 1) suggest possible differences in prophage stability by the CI repressor. To test this hypothesis, we measured the amount of cI transcripts when the prophage was integrated near the origin of replication (84.19′), in both orientations, using smFISH. Notably, the mean transcript numbers in both orientations were not significantly different from those observed when the prophage was integrated at the wild-type site (Fig. S1C).

### Bacterial growth in mixed cultures of lysogens with opposite integration orientation

**Fig 3:**
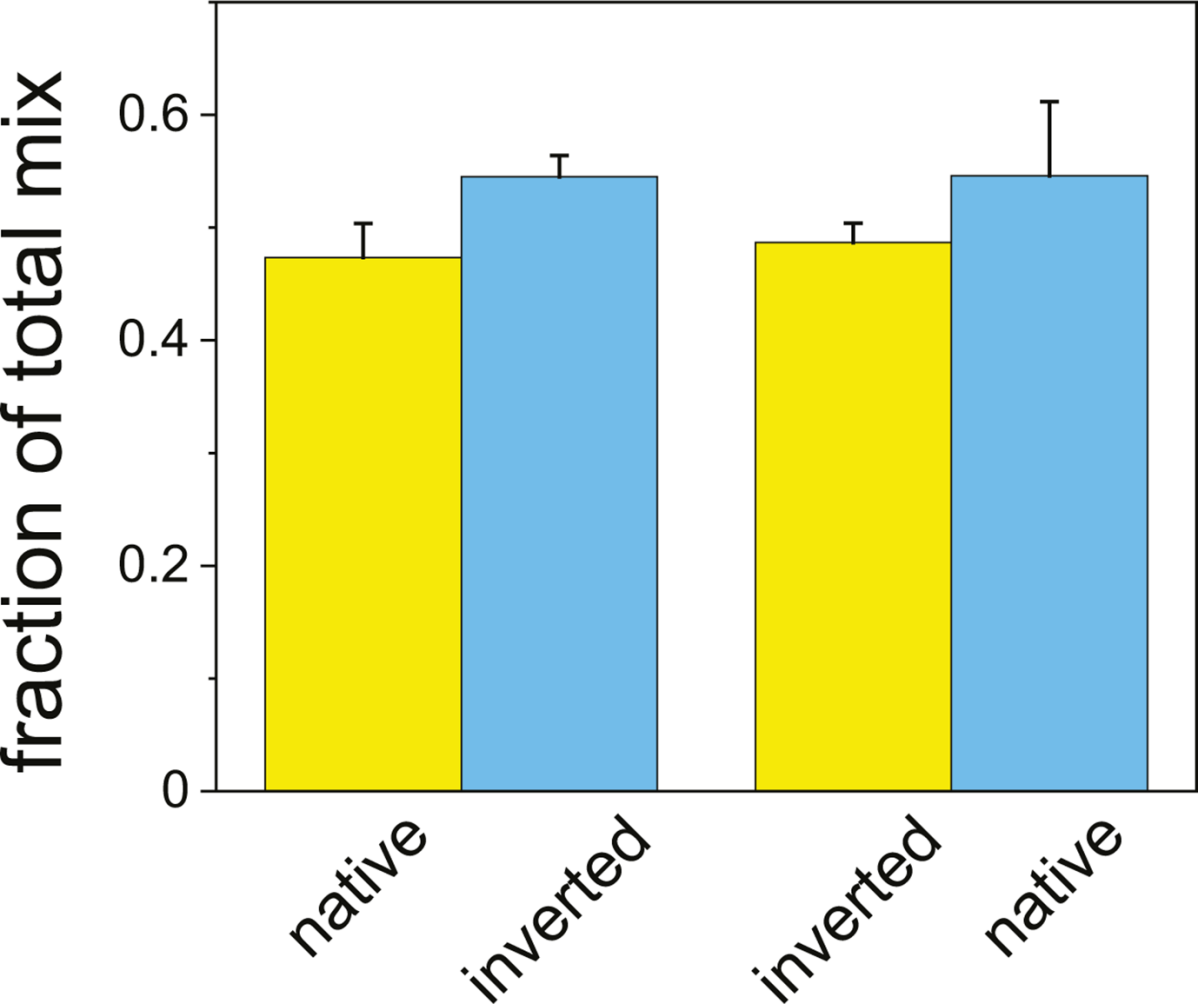
Measurements of bulk co-cultures of lysogen strain pairs with opposite orientation of integration at the wild-type *attB* site. Flow cytometry of lysogenic *E. coli* strains with lambda prophage integrated at the wild-type *attB* site (at 17.4′). Mixed cultures (1:1) with the prophages in the opposite integration orientations were grown for 14 days with daily dilution. Left two columns: a co-culture of strains with native and inverted prophage orientations labeled with YFP and CFP (XTL928/XTL925), respectively. Right two columns: strains with inverted and native prophage orientations labeled with YFP and CFP (XTL926/XTL927), respectively. Yellow and blue colors represent YFP and CFP labels, respectively. Error bars represent the standard deviation of four measurements.

To study whether there are growth differences in one lysogen orientation of integration over the other, we grew one strain and its inverted counterpart together for 14 days (Fig. 3), labeling each with either cyan fluorescent protein (CFP) or yellow fluorescent protein (YFP). Both fluorescent proteins were induced by the addition of arabinose and detected by flow cytometry. Irrespective of the labeling, no significant difference in growth was found, as shown in Fig. 3.

### Range expansions of lysogens with opposite integration orientation

**Fig 4:**
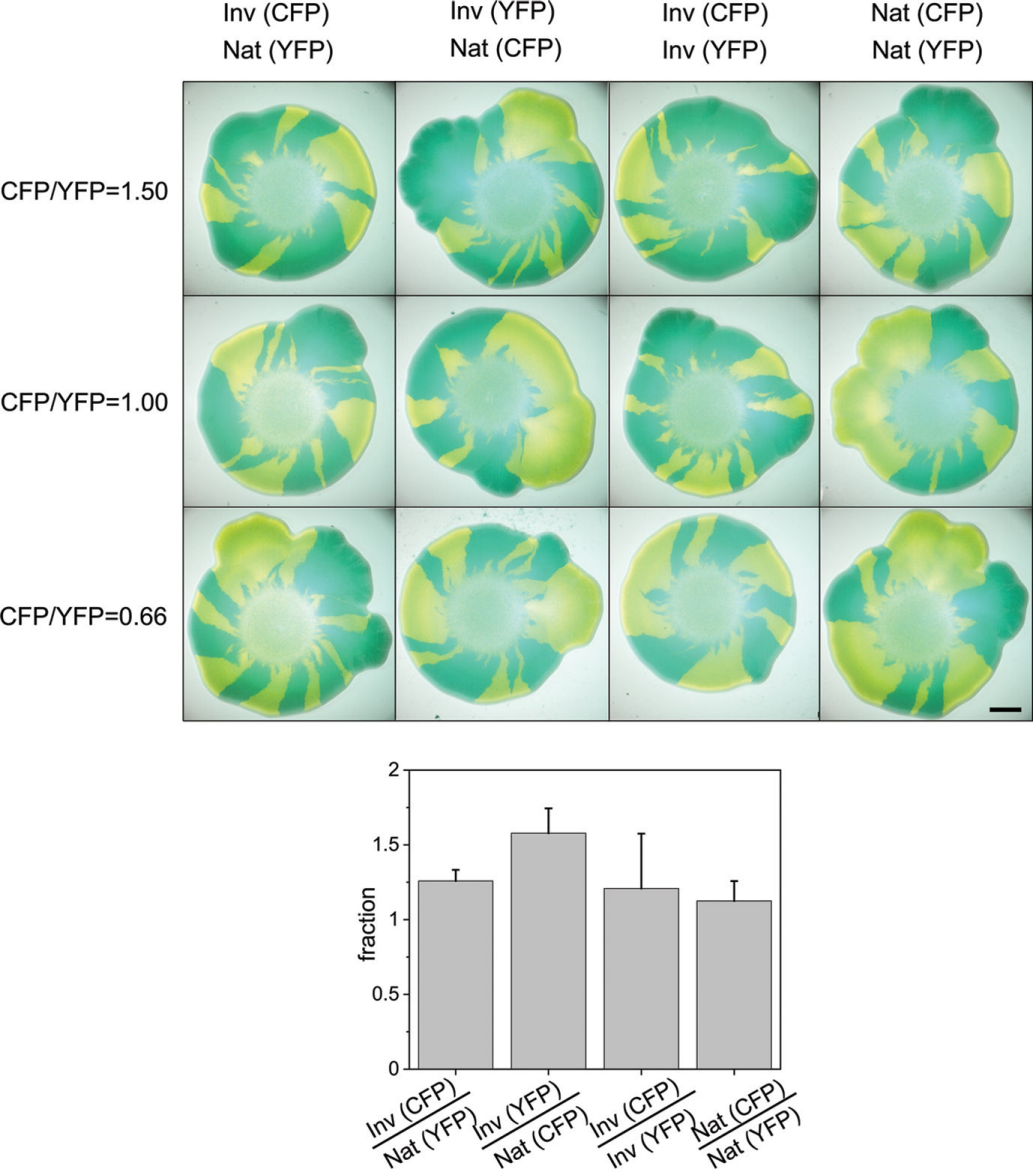
Range expansions of lysogenic strain pairs with prophages integrated at the wild-type site (17.4′) in various orientation combinations. Colony growth from a central region referred to as a homeland, comprising mixtures of bacteria: inverted (Inv) and native (Nat) integration orientations labeled with CFP and YFP (XTL925/XTL928), respectively; inverted and native orientations labeled with YFP and CFP (XTL926/XTL927), respectively; inverted-inverted orientations labeled with CFP and YFP (XTL925/XTL926), respectively; and native-native orientations labeled with CFP and YFP (XTL927/XTL928), respectively. Each image was recorded 3 days after seeding. Scale bar: 2 mm.

Range expansions are exquisitely sensitive to small differences in fitness due to the number fluctuations of reproducing pioneers within a region of small width at the expanding frontier (27). The measurements described above probed differential lysogen stability at the beginning (statistics of *cI* transcription) and at the very end (amount of free phage resulting from spontaneous induction) of the phage cycle after cell infection. To test mild differences between strains with phage integrated at the wild-type and symmetric sites (17.4′ and 50.6′, respectively) as well as in different directions relative to replication, we first carried out range expansions in mixed colonies of the wild-type strain and its inverted counterpart by labeling each strain with either CFP or YFP, whose production was induced by arabinose 1 day before images were recorded. In Fig. 4, we show the images of representative range expansions of mixed colonies seeded in different ratios and monitored with a stereomicroscope for 3 days. Growth beyond the initial homeland region led to well-defined sectors that flared out radially and whose overall area ratios were rather similar (starting from a mixture of cultures in a 1:1 ratio, the ratio of the inverted to native orientations was 1.4 ± 0.2, or 58% ± 4% of the area was covered by the strain with inverted prophages). Within each sector, the color was uniform, indicating that the sectors indeed represent populations with either one of the two prophage orientations. Note that when competing in a range expansion colony, phages corresponding to a given integration direction released after lysis cannot infect lysogens bearing prophages integrated in the opposite orientation since all strains carry *lamB* mutants and are resistant to lambda infection. Next, we carried out range expansion measurements to compare lysogenic strains with the wild-type attachment locus (17.4′) to its symmetric counterpart at 50.6′. Our results show that the strain with *attB* located at the wild-type site covered a smaller relative area, irrespective of the integration orientation. Starting from a mixture of cultures in a 1:1 ratio in the homeland, the percentage of area covered by the strain with a prophage integrated at the wild-type locus relative to a prophage integrated at the symmetric site was 33% ± 2% when the prophages were integrated in the direction orientation as replication (native), and 13% ± 4% when prophages were integrated in the inverted orientation (Fig. S2).

## DISCUSSION

The lysogenization of temperate phages and the subsequent adaptation of prophages in host chromosomes are potent drivers of genetic diversity in bacteria (8). In the present work, we studied the effects of changing both the integration orientation and the location of the integration site of bacteriophage lambda along the *E. coli* chromosome on the stability of the prophage state in order to understand the observed propensity of lambdoid prophages to co-orient with the direction of replication and on the non-uniform distribution of prophages along the *E. coli* chromosome, as described previously by Campbell (8, 11).

We start by discussing the effects of inverting prophage lambda at different locations along the bacterial chromosome. The observed co-orientation propensity of prophages with replication has been proposed as a mechanism to avoid the detrimental effects caused by replication-transcription collisions (31). Such a mechanism may inhibit the transcription of prophage genes, in particular, the *cI* repressor gene, which could lead to the induction of the lytic pathway. The 10-fold difference in speeds between DNA and RNA polymerases makes the difference between replication-transcription collision rates in both orientations minor, suggesting that this mechanism is unlikely. The small difference in the rate of replication-transcription collisions between the two directions cannot account for the observed preference in the orientation of integration of lambdoid prophages. The prolonged competition in bulk cultures and range expansion experiments in the present study show that inversion of integration orientation does not lead to significant changes in growth, consistent with the behavior reported originally by Campbell (9), who claimed that inversion of lambda prophages does not cause strong phenotypes in terms of bacterial growth or genetic instability (11). Indeed, lysogens bearing prophages in both orientations grew in general at the same rates, both in culture as well as in the range expansion assays. Colony roughness, the small number of sectors, and the strong spatial segregation that is observed in range expansion assays are consistent with a symmetric interaction between lysogenic strains with oppositely orientated prophages. The genetic drift due both to the randomness in cell division and lysis events at the small layer comprising the front of the expanding colony is amplified (27). The small effective population size in this layer gives rise to a continual bottleneck that causes strong segregation at the edge and the well-defined domains that we observed. Despite their sensitivity, range expansion assays did not reveal detectable growth differences when the lambda prophage was inverted at the wild-type *attB* site at 17.4′. Consistently, in these same two strains, differences in orientation of the prophage did not affect stability as measured by neither transcriptional bursts from the lambda *cI* repressor gene nor from phage release, suggesting similar CI repressor levels for both orientations. It is noteworthy that no change in transcription was detected for the *galK* gene when inverting the gal operon, despite the fact that transcription of both *galK* and *cI* is repressed by high-order structures (18, 32). The measured transcript number histograms and their statistical characteristics are comparable to the results previously reported (25, 33).

To shed light on the co-evolutionary selection of the location of *attB* along the chromosome by lambda phage and its *E. coli* host, we moved *attB* to different sites. We found that the effects on phage release due to spontaneous lysis are strongly position-dependent: notably, phage release at the wild-type site 17.4′ was maximal and about fivefold larger than when *attB* was located at the symmetric locus 50.6′. Furthermore, a decrease of more than two orders of magnitude in phage release was observed when *attB* was located near the origin of replication at 84.19′. We hypothesize that the significant differences in lysogen stability between the wild-type *attB* site and the other sites may be due to differences in chromosomal context affecting repression by CI or other mechanisms such as prophage integration/excision (34). Presumably, the regulation of phage release may be determined by local chromosomal context (35) near the *attB* site sequences flanking a prophage. One may speculate that the distance of the *cI* gene from the *attB* sites attenuates it from the effects of the chromosomal context. Consistently, the mean levels of cI transcripts at the wild-type site and near the origin of replication were found to be similar, in spite of the fact that the copy number of genes near the origin should be higher (36). However, experiments in which GFP was expressed from the *lac* promoter at various locations along the chromosome showed large variation in transcription caused by local chromosomal structure, in particular, near the origin of replication (37). Therefore, various factors in addition to gene dosage, e.g., local DNA packing and the collisions between transcription and DNA replication (38), can impact gene expression. Sequencing of the prophage in the flanking regions near the attachment sites excluded the possibility that the differences in phage release we observed between integration sites and orientations are due to mutations.

We found that inversion of prophage orientation near the origin of replication led to a significant reduction in free phage produced by spontaneous lysis, relative to more distal prophage at other locations. A reduction in phage numbers was observed when the SOS response was induced by mitomycin C for prophage located near the origin of replication. The effects of moving the attachment site to different locations on the chromosome on integration efficiency of lambda phage DNA have been studied previously (39), motivated by the fact that prophages are more abundant in regions closer to the terminus of replication (8). It was shown that near the origin of replication, the frequency of lysogenization was about twofold higher than at the wild-type *attB* location at 17.4′ or its symmetric counterpart, and even higher near the terminus. Hence, the conserved *attB* location suggests that the choice of lysis or lysogeny and the frequency of integration are rather independent.

Taken together, the inversion of the orientation direction of prophages was not observed to lead to detectable advantages in cell growth in our experiments, as measured with sensitive approaches including competition assays and range expansions. Yet, one may hypothesize that interaction with a host may play a role in the selection of integration orientation, as shown for DNA inversion states in microbiota in the presence of other bacteriophages, e.g., driven by phase variation (40, 41). We posit that the preference for a given orientation was set as a result of differences in prophage stability in a location-dependent way. Given that more than 80% of bacteria have been estimated to bear prophages in their chromosomes (42, 43), it would be worthwhile to evaluate to what extent the present conclusions apply to the stability of other temperate phages, including those of Gram-positive bacteria (44).

## MATERIALS AND METHODS

### Bacterial strains and plasmids

Standard genetic methods including recombineering and P1 transduction were used for strain construction (45). All strains were developed from an MG1655 wild-type strain. The temperature-sensitive plasmid pSIM18 that carries the *Red* genes, which provides recombination functions and confers hygromycin resistance, has been transformed into strains and their derivatives for recombineering purposes.

### Generation of strains with rearrangements of sites (*attB*) for the integration of bacteriophage lambda by recombineering

A schematic representation of the construction of the different strains in Table S1 is displayed in Fig. S3. The sequences of DNA oligomers used in the constructions are displayed in Table S2A. The pSIM18 was transformed into *E. coli* MG1655 to generate strain XTL336. This strain served as the base for the subsequent procedures.

### Deletion of the wild-type *attB* sequence

To construct strains with a non-native integration site, a strain lacking the *attB* sequence was created. First, a *tet-sacB* cassette was inserted into *attB* in strain XTL336 (MG1655/pSIM18) by recombineering after cassette amplification from T-SACK strain (45) using primers XT833 and XT834 and inserted into the *attB* WT site of strain XTL336 to generate a tetracycline-resistant XTL840 strain. Then, the single-stranded oligo XT835 was used to remove the *tet-sacB* cassette and *attB* site by recombineering (45), yielding strain XTL841.

### Native and inverted *attB* sequences in the wild-type integration site located at position 17.4′

The oligo XT836 was introduced into strain XTL840 to replace the *tet-sacB* cassette with an inverted *attB* sequence by recombineering. Selection of strain carrying the inverted *attB* was done using 6% sucrose counter-selection as previously described (45). Next, pSIM18 was removed by incubation at 37°C to generate XTL844. Similarly, the strain XTL 336 was used to generate XTL845, which has the *attB* sequence with a native orientation.

### Placement of the *attB* sequence at positions 34.05′, 50.6′, and 84.19′

To construct strains with an *attB* sequence at different chromosomal positions, a similar procedure was carried out as for the 17.4′ site. Primers XT849-XT850 (34.05′), XT857-XT858 (50.6′), and XT843-XT844 (84.19′) were used to amplify the *tet-sacB* cassette from *E. coli* T-SACK by PCR. The PCR products were inserted in their respective targets by recombineering in strain XTL841 and were selected by tetracycline. The *attB* sequence was then inserted in each site in either the native (same direction of the replication fork) or inverted orientations using the XT851-XT853, XT859-XT860, and XT845-XT846 primers, respectively. Integration sites were chosen in intergenic regions where there are no *E. coli* essential genes. In the lambda genome, the *b* region is adjacent to the lambda integration site and carries the *tI* terminator. In the prophage state, this strong terminator is then located adjacent to the bacterial DNA, preventing bacterial transcription from entering the *b* region of the prophage. Bacterial transcription from the other side of the integrated prophage is very unlikely to activate any of the lambda genes in this region and the stability of repression. Strains were selected by sucrose counter-selection, and the pSIM18 plasmid was removed by incubation at 37°C.

### Confirmation of single-copy integration in lambda lysogens

To confirm single-copy integration, colony PCR was carried out as described previously (46). Infected strains (e.g., XTL844 and XTL845) with wild-type lambda phage and isolate cells that have become immune to further infection by lambda were tested for multiple lambda prophage integration. Primers powell_1, powell_2, and powell_3 were used in colony PCR for confirmation. For single-copy inverted integration in strain XTL848, the expected PCR bands should be 221 bp, and for multiple-copy integration, it should be 221 and 379 bp. For native orientation in strain XTL850, the PCR bands of single-copy integration should be 501 bp, and for multiple-copy integration, it should be 501 and 379 bp (see sequences in Table S2B) (46). Similar confirmation in other strains was performed at other integration sites.

### Bacterial genome sequencing

Bacterial overnight cultures of the strains in Table S3 were diluted 1:100 and then grown to OD_600_ = 0.4–0.6, and then 4 mL was pelleted at 6,000 × *g* for 10 min. The Qiagen DNeasy Blood and Tissue Kit was used for bacterial genomic DNA extraction, following the manufacturer’s protocol for Gram-negative bacteria. The genomic DNA was then sequenced by long-read sequencing technology (Oxford Nanopore Technologies) through Plasmidsaurus sequencing service. This service provides whole-genome sequencing and assembly of genomic DNA from a clonal population of bacteria. The presence of the prophage sequence was verified by Blastn sequence searches, which queried each nucleotide assembly against the nucleotide sequence of *Escherichia* phage Lambda (cI857), using NCBI BLAST+ (2.10.0) software. Blast hits with >99% sequence identity were documented, and their bacterial genomic neighborhood was inspected using IGV software (Integrative Genomics Viewer, the Broad Institute) to identify the prophage integration orientation as well as single-copy integration (Table S3). The regions flanking prophage integration sites are shown in Fig. S4.

### Knockout lamB and insertion of pBAD-CFP and pBAD-YFP into the strains by P1 transduction

To avoid binding of free phages to cell debris, as this affects plaque assays, the *lamB* gene was deleted and replaced with *CAT* by P1 transduction (43) in all strains carrying the lambda prophage, yielding strains XTL855, XTL856, XTL891, XTL892, XTL893, XTL894, XTL905, and XTL906. For labeling, a construct with the arabinose promoter P_BAD_, ampicillin resistance, and either the YFP or the CFP was inserted by P1 transduction replacing the arabinose operon *araBAD*. Both fluorescent proteins were transduced into strains XTL855 and XTL856. Similarly, fluorescent-labeled strains were constructed at the site symmetric to the wild type (50.6′) (Table S1). Sequencing confirmed that strains XTL855, XTL856, XTL893, XTL894, XTL862, and XTL863 do not carry the *lamB* gene.

### Growth media and conditions

Bacterial strains as indicated were propagated in LB medium at 37°C overnight from a single colony inoculum. For experiments, cultures were diluted 1:100 into fresh LB medium and grown to an OD_600_ of 0.2–0.4 before the indicated treatment. Cultures were then diluted 1:100 into LB medium with different concentrations of arabinose, as indicated, and allowed to grow for about 3 hours to OD_600_ of 0.4–0.6. Under these conditions, cells were in a state of exponential growth (Table S4; Fig. S5). Tetracycline (12.5 µg/mL), hygromycin (200 µg/mL), chloramphenicol (10 µg/mL), and ampicillin (30 µg/mL) were added as needed.

### Single-molecule fluorescence *in situ* hybridization

Bacterial cultures were grown and treated as indicated above. Single-molecule fluorescence *in situ* hybridization experiments were performed following previously published procedures (25) with the indicated modifications (26, 47). The design of smFISH probes was carried out using the Probe Designer algorithm (Stellaris, Biosearch Technologies); the labeled sequences of smFISH probes of the indicated genes are listed in Table S5. Images were taken on a Nikon Eclipse–Ti-E microscope controlled by the NIS-Elements software using a 100× N.A 1.45 oil immersion phase contrast objective lens (Nikon plan-apochromat 100× 1.45 lambda) and an Andor iXon X3 EMCCD camera. All the filters are from Chroma. The filters used were ET-534/30× for excitation, 560lp as a dichroic mirror, and ET-585/40M for the emission. A phase contrast image was acquired followed by a z-stack of 13 slices and 250 nm spacing of fluorescence images with 2 s integration time of each slice. Each sample was imaged at multiple locations to get a total of at least 500 cells.

### Image and smFISH data analysis

Spot recognition was performed as described previously (26). Single-cell recognition is based on the MicrobeTracker suite (48), which was integrated into our custom MATLAB program. To quantify the fluorescence intensity of each mRNA spot and correct for variation in cell labeling, the following procedure was used: the average intensity from a diffraction-limited spot was measured. We then subtracted from the intensity of each spot in a bacterium the median intensity value of the pixels in this bacterium that did not contain a fluorescent spot (47). The variation of the mRNA numbers between experiments is about 20%, which is comparable to previous works (25). For each histogram defined by *m*_*i*_ (the midpoint of *i*th bin), the bin frequency *n*_*i*_, and total sample size, the standard deviation is defined by $SD=\sqrt{\sum n_{i}{\left(m_{i}-\mu \right)}^{2}/\left(N-1\right)}$, where $\mu =\sum m_{i}n_{i}/N$ (gstd function in Matlab). ${SD}_{d}$ represents a mean over independent runs.

### Release of lambda phage from the prophage state in untreated or mitomycin C-induced cultures

A single colony of the indicated strain was grown in LB medium at 37°C overnight. The culture was then washed twice and centrifuged at 8,143 × *g* for 7 min. The pellet was then suspended in the same volume of Tris magnesium glycerin (TMG) buffer as the original inoculum, diluted 400-fold (30 µL of the cells into 12 mL of LB broth), and incubated in 37°C water bath shaker at 220 rpm. At an OD_600_ of 0.1, cultures were either untreated or induced by mitomycin C (1 µg/mL). Incubation was continued until the cells lysed (about 3 hours), and then 200 µL of chloroform was added. Under these conditions, the concentration of mitomycin C was saturating. Cell debris was spun down at 8,143 *× g* for 7 min, and supernatant lysate containing phage was saved. Lysates were diluted in TMG, and overnight culture of MG1655 bacteria in LB was infected (100 µL) and incubated on ice for 30 min to adsorb phage by cells. Infected cells were added to 2.5 mL of molten TB top agar (0.6%) and immediately poured onto TB agar 10 cm plates. The agar plates were incubated at 37°C overnight, and the number of plaques was recorded. The normalized plaque number per milliliter to the OD_600_ of cells when treated with mitomycin C was then calculated. For lambda phage spontaneous release from the prophage state, the indicated bacterial strains were treated as described above (release of lambda phage from the prophage state by mitomycin C induction) until the 400-fold diluted cultures reached an OD_600_ of about 0.2–0.3 in about 2 hours. Cultures were then spun down to generate a supernatant that contained phage generated from cells that spontaneously induced during growth. These lysates were passed through a 0.22 µm filter to remove any lysogenic cells still present. Normalized plaque numbers per milliliter with respect to the OD_600_ of cells were determined as above. Lambda virions are stable for weeks in the LB medium.

### Competition assays

The competition assay was derived from Chait et al. (49). Strains with lysogens integrated in opposite directions were labeled with either YFP or CFP on the chromosome, under an arabinose promoter. To ascertain that differences in fitness are not due to the fluorescent label, colors were also swapped between the lysogen-bearing strains (Table 1). Overnight cultures at OD_600_ = 0.4 diluted into M63 minimal medium supplemented with MgSO_4_ (1 mM), glucose (0.5%), Casamino Acids (0.1%), thiamine (1 mg/L), and standard concentrations of the appropriate antibiotics were grown at a 1:1 ratio in flat-bottomed, 96-well plates. The plates were grown in the dark at 30°C at 600 rpm on an orbital shaker for 10 days and diluted (1:100) in M63 medium containing supplements every 24 hours to maintain them at the logarithmic phase for about seven generations. Arabinose (0.5%) was added to the cultures on the last day. The number of cells expressing each fluorescently label protein was determined, and images of cells were taken by an Imaging Flow Cytometer (ImageStreamX Mark II, AMNIS corp.—part of Luminex, TX, USA). Data were acquired using a 60× lens (NA = 0.9); lasers used were 405 nm (120 mW), 488 nm (100 mW), and 785 nm (10 mW). Channels acquired were 1 and 9 for the brightfield, Ch2 for YFP, Ch6 for side scatter, and Ch7 for CFP. Data were analyzed using the manufacturer’s image analysis software IDEAS 6.3 (Amnis corp.). Images were compensated for spectral overlap using single-stained controls. Single events were selected by plotting the area of the cell (AREA_M01, in square micrometers) vs the aspect ratio (the minor axis divided by the major axis) of the bright field image. To eliminate out-of-focus cells, cells were further gated using the gradient RMS and contrast features (measures the sharpness quality of an image obtained by detecting large changes in pixel values in the image). Cells positive for YFP or CFP expression were selected by plotting the intensity vs max pixel (the value of the high intensity pixel) of the relevant channel.

### Range expansion experiments

Range expansion experiments were performed in 6-well plates (Catalog No. 3516, Corning, USA) with 3 mL of LB media and 1.5% agar per well. Plates were left to dry for about 3 days so that the drops did not spread over the agar surface. We compared pairs of strains with different orientations of the lambda prophage and with the same orientations but different insertion sites in the chromosome, expressing either CFP or YFP. For controls, we compared strains with the same genotype but different fluorescent markers. Strains were grown to an OD = 0.3 and mixed in 0.66:1, 1:1, and 1:1.5 volume ratios. We verified that the ratios measured by OD corresponded to real cell ratios by counting individual cell ratios per field of view using fluorescence microscopy. The mixtures were diluted 10-fold in PBS, and 2.5 µL was seeded in the center of a well. Plates were incubated at 37°C for 1–3 days. After incubation, a sterile piece of a stainless steel tube was used to cut a circle around the bacterial colonies, and then the agar around it was carefully removed with tweezers. For fluorescence induction, 500 µL of 50% arabinose solution was added to the carved-out space surrounding the colonies, and the plates were left incubating at room temperature overnight. Sixteen-bit black and white images were captured using an SMZ-25 stereoscope (Nikon, Japan) equipped with an Andor Zyla VSC-13476 camera (Oxford Instruments, UK), a SOLA Light Engine (Lumencor, USA), and the 49001-ET-ECFP and 49003-ET-EYFP filter sets (Chroma Technology Corporation, USA). Bright-field (exposure = 50 ms, no binning), CFP (exposure = 1,000 ms, 3 × 3 binning), and YFP (exposure = 500 ms, 3 × 3 binning) channels were recorded. The image, obtained by dividing the values of the first channel by those of the third one for better contrast (see Fig. S6, top panel, where dark regions correspond to the CFP-labeled strain), was analyzed using MATLAB. After choosing a region of interest (ROI), the outer ring of the two-strain colony, the image was thresholded (see Fig. S6, middle panel) and the despeckled ROI (using medfilt2) was false-colored (see Fig. S6, bottom panel). Finally, we computed the ratio of the blue-colored area to the yellow-colored area in the ROI.

## Data Availability

The raw data used image analysis tools to generate the figures, and the Nanopore sequence data are publicly available at https://datadryad.org/stash/share/N-O9D-Fs2X834XIZlwDeDYH93Rb9evQKU3UITVemwZ0.

## SUPPLEMENTAL MATERIAL

Supplemental Material mbio.02078-23-s0001.pdf Figures S1-S6; Tables S1-S5.

ASM does not own the copyrights to Supplemental Material that may be linked to, or accessed through, an article. The authors have granted ASM a non-exclusive, world-wide license to publish the Supplemental Material files. Please contact the corresponding author directly for reuse.
