# Supplementary material for: Stability and gene strand bias of lambda prophages and chromosome organization in Escherichia coli
Source: mBio. 2024 Jun 18;15(7):e02078-23. doi: 10.1128/mbio.02078-23 (PMC11253608; doi:10.1128/mbio.02078-23)
Supplement: Supplemental Material — Figures S1-S6; Tables S1-S5. [file mbio.02078-23-s0001.pdf]

SUPPLEMENTAL MATERIAL

Stability and gene strand bias of lambda prophages and chromosome organization in *Escherichia coli*

Xintian Li, Oscar Gallardo, Elias August, Bareket Dassa, Donald L. Court, Joel Stavans and Rinat Arbel-Goren

Figure S1. Transcript variability of native and inverted orientations of the gal operon in a wild-type background and cI in a *recA*⁻ background.

Figure S2. Schematic configuration of range expansion assays of lysogenic strains with prophages integrated at the wild-type site and its symmetric counterpart.

Figure S3. Strains developed and their construction scheme.

Figure S4. Bacterial genome sequencing alignment.

Figure S5. Bacterial growth curves.

Figure S6. Analysis of range expansion of a fluorescently-labeled lysogenic strains.

Table S1. *E. coli* strains

Table S2A. DNA oligomers used to construct the *E. coli* strains in Table S1.

Table S2B. DNA oligomers for verifying single copy prophages.

Table S3. Confirmation of the localization and orientations of prophage in different strains long-read sequencing.

Table S4. Doubling times of lysogenic strains.

Table S5. Sequences of smFISH probes.

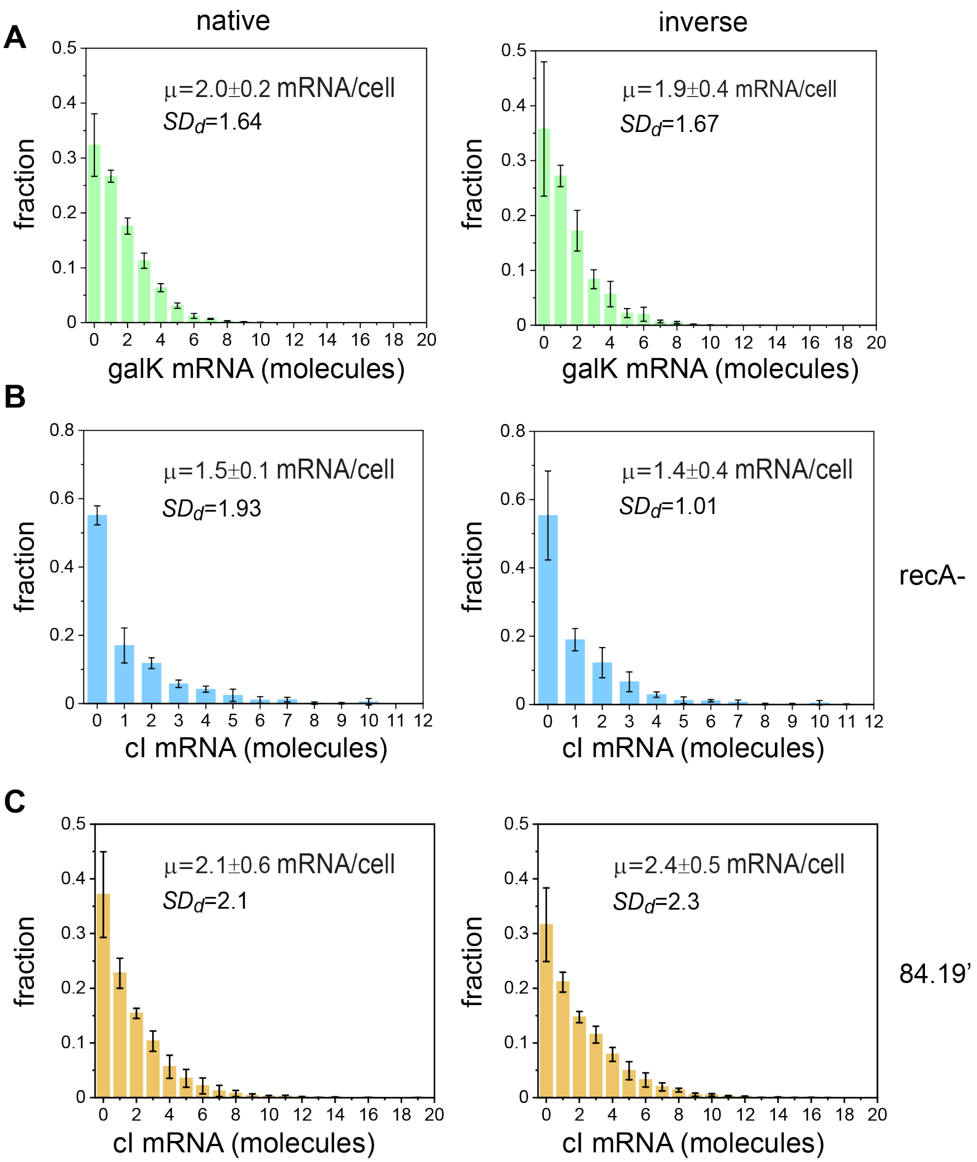

Figure S1. Transcript variability of native and inverted orientations of the gal operon in a wild-type background and cI in a *recA*⁻ background. (**A**) Distributions of galK transcripts in lysogens integrated in a native (left, XTL856) and inverted (right, XTL855) orientation. (**B**) Distributions of cI transcripts measured in *recA*⁻ lysogens integrated with a native (left) and inverted (right) orientations. The distributions, measured by smFISH, represent an average over three experimental repeats and error bars represent standard errors. (**C**) Distributions of cI transcripts measured in lysogens integrated at 84.19' with a native (left, XTL894) and inverted (right, XTL893) orientations. All distributions represent an average over at least three independent experimental repeats (about 3000 cells for each orientation), while error bars represent standard errors.

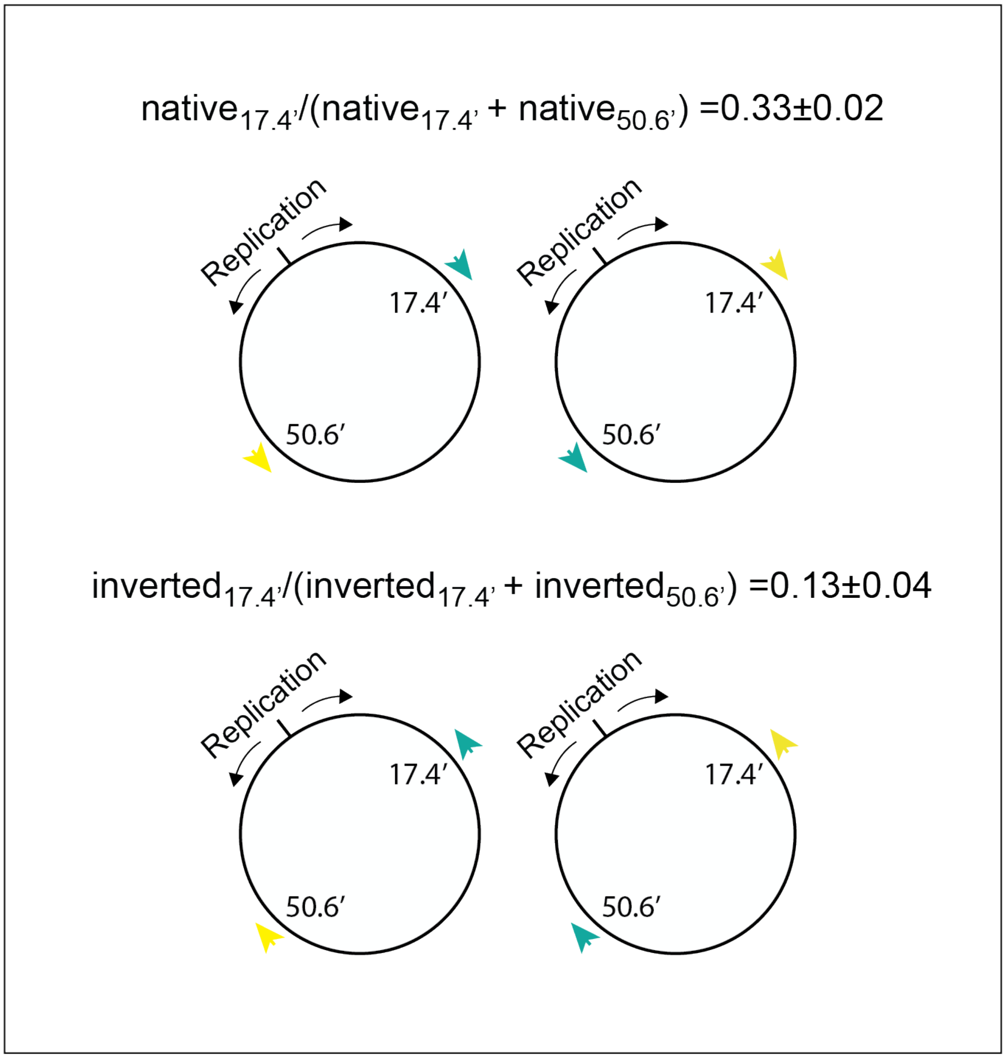

Figure S2. Schematic configuration of range expansion assays of lysogenic strains with prophages integrated at the wild-type site and its symmetric counterpart. Mixed bacterial colonies (ratio=1:1) of strain pairs with prophages at 17.4’ and 50.6’ sites, each with the prophage integrated either in inverted or native integration orientations, labelled with CFP and YFP. The ratio of area of the wild-type *attB* site from the total is shown, determined from two independent experiments, each consisting of nine colonies (mean ± standard error). The schemes represent the chromosomal constructions, the arrows, the prophage integration orientations relative to the replication direction and the blue and yellow colors of the arrows represent CFP and YFP, respectively.

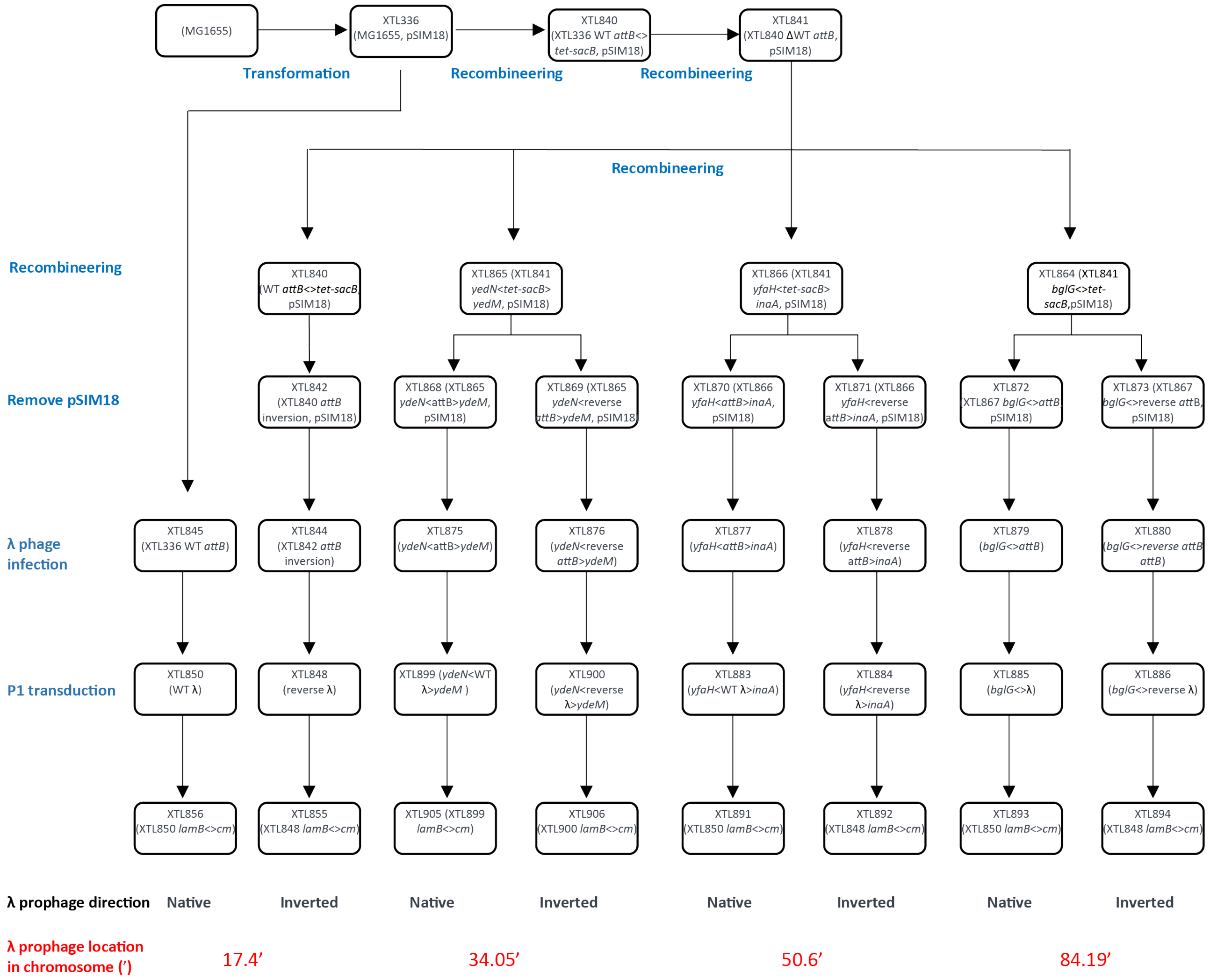

Figure S3. Strains developed and their construction scheme. The diagram shows the methods that were used in order to develop the indicated strains including: transformation, recombineering, lambda phage infection and P1 transduction (in blue). The indicated locations of integration at the *E. coli* chromosome and the phage orientations are indicated for each strain.

XTL862-XTL863

Near *ybhB*:

Prophage sequence

XTL862 TTTGACTGATAGTGACCTGTTCGTTGCAACAAATTGATAAGCAATGCTTTTTTATAATGCCAAC**TTAGTATAAAAAAG**

XTL863 TTTGACTGATAGTGACCTGTTCGTTGCAACAAATTGATAAGCAATGCTTTTTTATAATGCCAAC**TTAGTATAAAAAAG**

***E. coli* sequence**

XTL862 GAAAATGTGTTCACAGGTTGCTCCGGGCTATGAAATAGAAAAATGAATCCGTTAA-CAAG**TTAGTATAAAAAAGC**

XTL863 GAAAATGTGTTCACAGGTTGCTCCGGGCTATGAAATAGAAAAATGAATCCGTTAAGCCTGCTTTTTTATACTAA-

Near *ybhC:*

Prophage sequence

XTK862 **TTAGTATAAAAAAGC**TGAACGAGAAACGTAAAATGATATAAATATCAATATATTAAATTAGATTTTGCATAAAAAACAGACTACATAATA

XTL863 **TTAGTATAAAAAAGC**TGAACGAGAAACGTAAAATGATATAAATATCAATATATTAAATTAGATTTTGCATAAAAAACAGACTACATAATA

***E. coli* sequence**

XTL862 **TTAGTATAAAAAAGC**AGGCAGCGAAACGGGAAGGTAAAAAGACAAAAAGTTGTTTTTAATACCTTTAAGTGATACCAGATGGCATTG

XTL863 CTTTTTTATACTAACTTG-AGCGAAACGGGAAGGTAAAAAGACAAAAAGTTGTTTTTAATACCTTTAAGTGATACCAGATGGCATTG

XTL855 and XTL856

Near *ybhB*

Prophage sequence

XTL855 TATTTTGACTGATAGTGACCTGTTCGTTGCAACAAATTGATAAGCAATGCTTTTTTATAATGCCAAC**TTAGTATAAAAAAGC**

XTL856 TATTTTGACTGATAGTGACCTGTTCGTTGCAACAAATTGATAAGCAATGCTTTTTTATAATGCCAAC**TTAGTATAAAAAAGC**

***E. coli* sequence:**

XTL855 TGTGTTCACAGGTTGCTCCGGGCTATGAAATAGAAAAATGAATCCGTTGAA-CAAG**TTAGTATAAAAAAGC**

XTL856 TGTGTTCACAGGTTGCTCCGGGCTATGAAATAGAAAAATGAATCCGTTGAAGCCTGCTTTTTTATACTAA-

Near *ybhC*

Prophage sequence

XTL855 **TTAGTATAAAAAAGC**TGAACGAGAAACGTAAAATGATATAAATATCAATATATTAAATTAGATTTTGCATAAAAAACAGACTACATAATA

XTL856 **TTAGTATAAAAAAGC**TGAACGAGAAACGTAAAATGATATAAATATCAATATATTAAATTAGATTTTGCATAAAAAACAGACTACATAATA

***E. coli* sequence**

XTL855 **TTAGTATAAAAAAGC**AGGCAGCGAAACGGGAAGGTAAAAAGACAAAAAGTTGTTTTTAATACCTTTAAGTGATACCAGATGGCATTGCG

XTL856 CTTTTTTATACTAACTTG-AGCGAAACGGGAAGGTAAAAAGACAAAAAGTTGTTTTTAATACCTTTAAGTGATACCAGATGGCATTGCG

XTL893 and XTL894

Near *oriC*

Prophage sequence

XTL893 **TTAGTATAAAAAAGC**TGAACGAGAAACGTAAAATGATATAAATATCAATATATTAAATTAGATTTTGCATAAAAAACAGACTACATAATA

XTL894 **TTAGTATAAAAAAGC**TGAACGAGAAACGTAAAATGATATAAATATCAATATATTAAATTAGATTTTGCATAAAAAACAGACTACATAATA

***E. coli* sequence**

XTL893 CGTTCAGCTTTTTTATACTAA--CTTGATCCTCCATATTTCCGCTCATTTGGGCACTGACCAGATGCATGGCAATAAAGCCCACTTCA

XTL894 AATGCCAAC**TTAGTATAAAAAAGC**AGGATCCTCCATATTTCCGCTCATTTGGGCACTGACCAGATGCATGGCAATAAAGCCCACTTCA

Near *bglF*

Prophage sequence

XTL893 ACTGATAGTGACCTGTTCGTTGCAACAAATTGATAAGCAATGCTTTTTTATAATGCCAAC**TTAGTATAAAAAAGC**

XTL894 ACTGATAGTGACCTGTTCGTTGCAACAAATTGATAAGCAATGCTTTTTTATAATGCCAAC**TTAGTATAAAAAAGC**

***E. coli* sequence**

XTL893 AATTTTATTAATTGCAGCATTTCGCGCATTAACTGCGTGACACCTGCAACCCTGCTTTTTTATACTAAGTTGGCATTATAAAAAA

XTL894 AATTTTATTAATTGCAGCATTTCGCGCATTAACTGCGTGACACCTGCAACCAAG--**TTAGTATAAAAAAGC**TGAACGAGAAACGT

The nucleotide sequence at the prophage integration sites for all the sequenced strains was identical for the corresponding orientation: AAGTTAGTATAAAAAAGC.

Figure S4. Bacterial genome sequencing alignment. Bacterial strain sequences of both integration orientations obtained from Nanopore sequence assemblies aligned near the flanking regions to the indicated bacterial genes. Gray shaded regions denote prophage sequences while the integration sites are shown in bold.

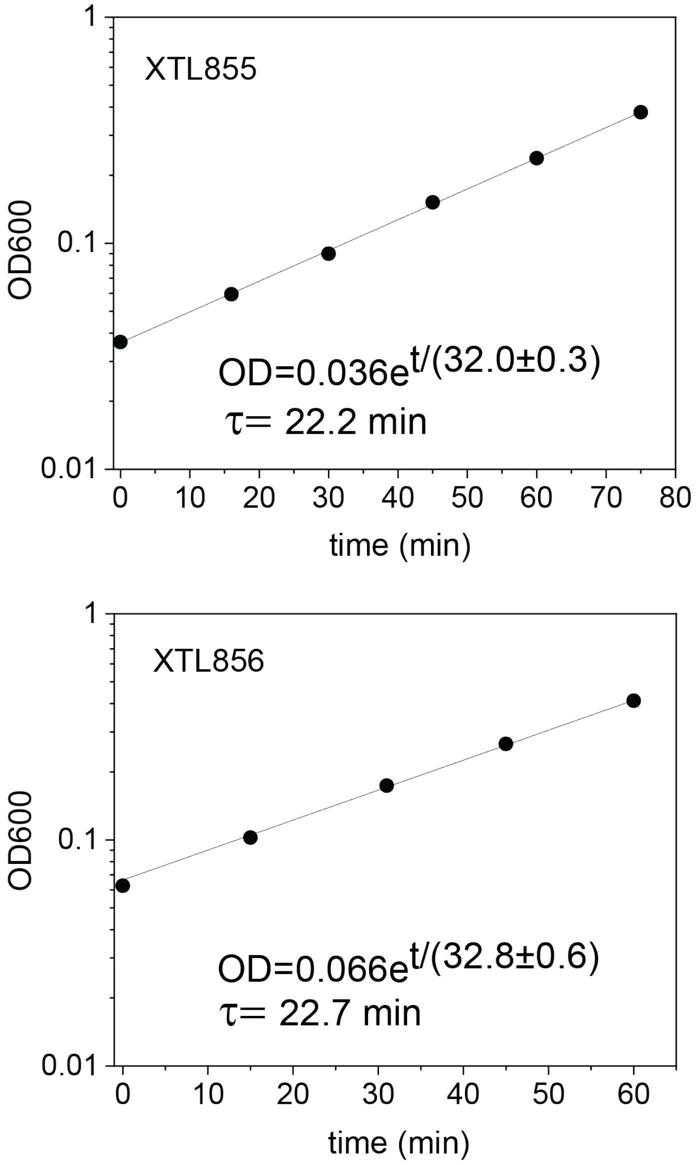

Figure S5. Bacterial growth curves. The growth in logarithmic scale of strains XTL855 and XTL856 in LB was followed using optical density (OD_{600}). The solid line represents an exponential fit to the data and the doubling times (τ) corresponding to the time constants of the fits are shown. The doubling time of all other strain in the study were determined similarly and are shown in Table S3.

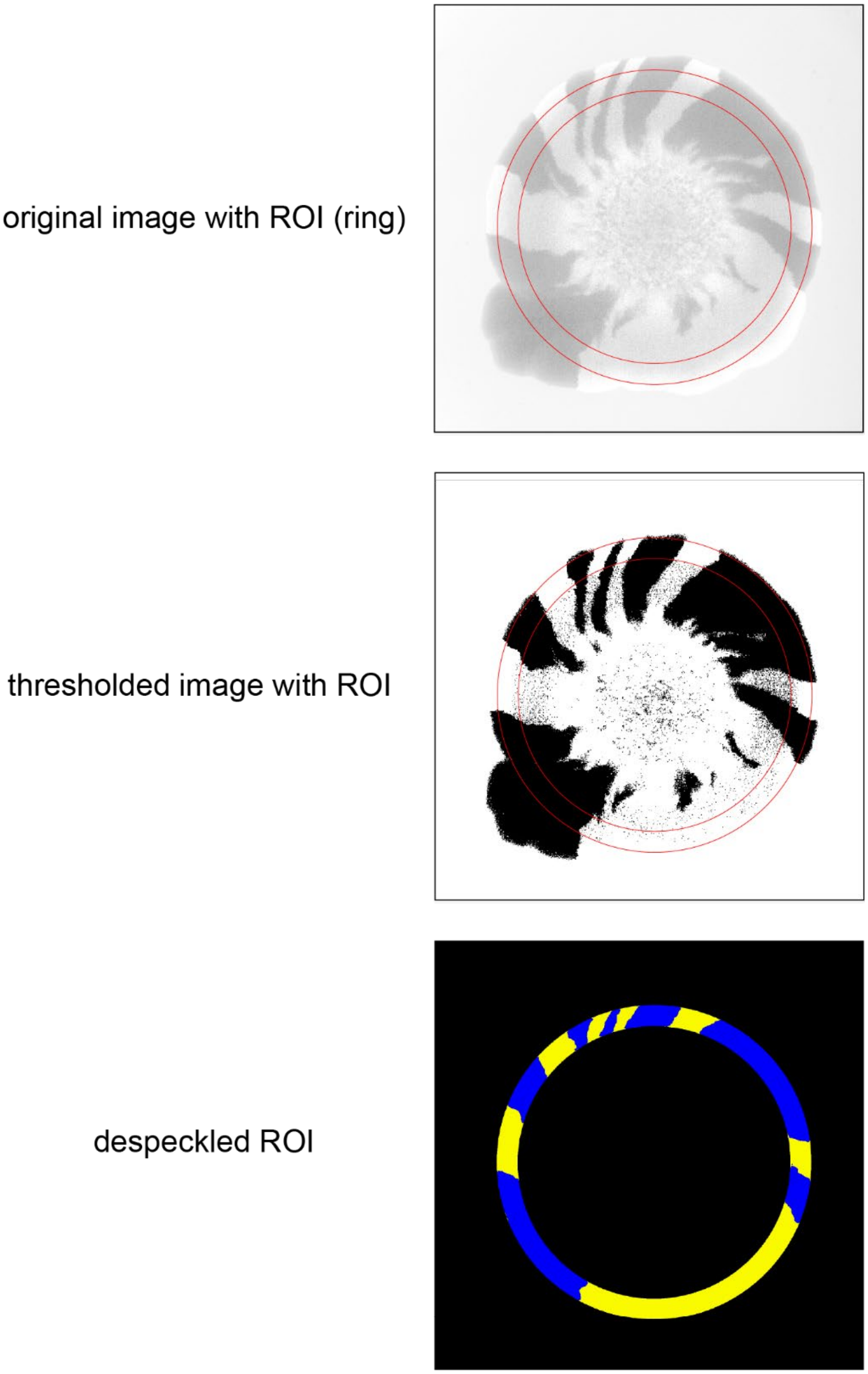

Figure S6. Analysis of range expansion of a fluorescently-labeled lysogenic strains. An exemplary range expansion image (top) with Region Of Interest (ROI) is shown, where dark regions correspond to the CFP-labeled strain. After achieving better contrast (see main text), the image threshold was subtracted (middle) and the despeckled ROI was false-colored (bottom).

Table S1. ***E. coli*** **strains**

**Strain**	**Description**
MG1655	Wild-type
XTL336	MG1655+pSIM18
XTL840	XTL336 *attB<>tet-sacB*. XTL336 recombineering with PCR product (XT833 XT834 PCR *T-SACK*), selected with tet plate.
XTL841	XTL840 *attB* ΔpSIM18. XTL336 recombineering with XT835, selected with sucrose plate.
XTL842	XTL840 *attB* inversion pSIM18. XTL336 recombineering with XT836, selected with sucrose plate.
XTL844	XTL842 *attB* inversion. XTL842 ΔpSIM18 by grow in 37 ^{o}C, Hygro sensitive.
XTL845	MG1655. XTL336 ΔpSIM18 by grow in 37 ^{o}C, Hygro sensitive.
XTL848	XTL844 Single copy of inverted λ phage integration, λ prophage at 17.4'. XTL844 infected with λ phage and test single copy.
XTL850	XTL845 Single copy of wild-type direction λ phage integration, λ prophage at 17.4'. XTL845 infected with λ phage and test single copy.
XTL855	XTL848 *lamB<>cat*, λ prophage at 17.4'. XTL848 P1 transduction with XTL846 (*lamB<>cm*)
XTL856	XTL850 *lamB<>cat*, λ prophage at 17.4'. XTL850 P1 transduction with XTL846 (*lamB<>cm*)
XTL862	XTL855 recA::tn10. XTL855 P1 transduction with recA::tn10 lysis, selected with tet cit plate. MG1655 inverted λ phage integration, *lamB<>cm*, *recA*::*tn10*
XTL863	XTL856 recA::tn10. XTL856 P1 transduction with recA::tn10 lysis, selected with tet cit plate. MG1655 wild-type direction λ phage integration, *lamB*<>cm, *recA*::*tn10*
XTL864	XTL841 *bglG<>tet-sacB* (84.19'). XTL841 recombineering with PCR product (XT843 and XT844 PCR *T-SACK*), selected with tet plate. pSIM18 lost
XTL865	XTL841 *ydeN<tet-sacB>ydeM* (34.05'). XTL841 recombineering with PCR product (XT849 and XT850 PCR *T-SACK*), selected with tet plate.
XTL866	XTL841 *yfaH<tet-sacB>inaA* (50.6'). XTL841 recombineering with PCR product (XT857 and XT858 PCR *T-SACK*), selected with tet plate and test the sucrose sensitive.
XTL867	XTL864 pSIM18. XTL864 transform with pSIM18 plasmid, selected with Hygromycin plate.
XTL868	XTL865 *ydeN<attB>ydeM* (34.05'). XTL865 recombineering with oligo XT851, selected with sucrose plate.
XTL869	XTL865 *ydeN*<inverted *attB>ydeM* (34.05'). XTL865 recombineering with oligo XT853, selected with sucrose plate.
XTL870	XTL866 *yfaH<attB>inaA* (50.6'). XTL866 recombineering with oligo XT859, selected with sucrose plate.
XTL871	XTL866 *yfaH*< inverted *attB>inaA* (50.6'). XTL866 recombineering with oligo XT860, selected with sucrose plate.

Strain	Description
XTL872	XTL867 *bglG*<>*attB* (84.19'). XTL867 recombineering with oligo XT845, selected with sucrose plate.
XTL873	XTL867 *bglG*<> inverted *attB* (84.19'). XTL867 recombineering with oligo XT846, selected with sucrose plate.
XTL875	XTL868 ΔpSIM18. Total: MG1666 Δwild-type *attB*, *ydeN*<attB>*ydeM* (34.05')
XTL876	XTL869 ΔpSIM18. Total: MG1666 Δwild-type *attB*, *ydeN*< inverted *attB*>*ydeM* (34.05)
XTL877	XTL870 ΔpSIM18. Total: MG1666 Δwild-type *attB*, *yfaH*<*attB*>*inaA* (50.6')
XTL878	XTL871 ΔpSIM18. Total: MG1666 Δwild-type *attB*, *yfaH*< inverted *attB*>*inaA* (50.6')
XTL879	XTL872 ΔpSIM18. Total: MG1666 Δwild-type *attB*, *bglG*<>*attB* (84.19')
XTL880	XTL873 ΔpSIM18. Total: MG1666 Δwild-type attB, *bglG*<> inverted *attB* (84.19')
XTL883	XTL877 λ prophage integration at 50.6'
XTL884	XTL878 λ prophage integration at 50.6'
XTL885	XTL879 λ prophage integration at 84.19'
XTL886	XTL880 λ prophage integration at 84.19'
XTL891	XTL883 *lamB*<>*cm*, λ prophage at 50.6'. XTL883 P1 transduction with XTL846 P1 lysis, selected with cm cit plate.
XTL892	XTL884 *lamB*<>*cm*, λ prophage at 50.6'. XTL884 P1 transduction with XTL846 P1 lysis, selected with cm cit plate.
XTL893	XTL885 *lamB*. XTL885 P1 transduction with XTL846 P1 lysis, selected with cm cit plate.
XTL894	XTL886 *lamB*. XTL886 P1 transduction with XTL846 P1 lysis, selected with cm cit plate.
XTL899	XTL875 λ prophage integration at 34.05', single copy.
XTL900	XTL876 λ prophage integration at 34.05', single copy.
XTL901	XTL899 pSIM18.
XTL902	XTL900 pSIM18.
XTL905	XTL901 *lamB*<>*cm*. XTL901 P1 transduction with XTL846 P1 lysis, selected with cm cit plate at 37 ^{o}C.
XTL906	XTL902 *lamB*<>*cm*. XTL902 P1 transduction with XTL846 P1 lysis, selected with cm cit plate at 37 ^{o}C.
XTL925	XTL855 P_{ara} *cfp-amp*. P1•IRE111 P_{ara} *cfp-amp* into XTL855

Strain	Description
XTL926	XTL855 P_{ara} *yfp-amp*. P1·IRE112 P_{ara} *yfp-amp* into XTL855
XTL927	XTL856 P_{ara} *cfp-amp*. P1·IRE111 P_{ara} *cfp-amp* into XTL856
XTL928	XTL856 P_{ara} yfp-amp. P1·IRE112 P_{ara} *yfp-amp* into XTL856
XTL929	XTL891 P_{ara} *cfp-amp*. P1·IRE111 P_{ara} *cfp-amp* into XTL891
XTL930	XTL891 P_{ara} *yfp-amp*. P1·IRE112 P_{ara} *yfp-amp* into XTL891
XTL931	XTL892 P_{ara} *cfp-amp*. P1·IRE111 P_{ara} *cfp-amp* into XTL892
XTL932	XTL892 P_{ara} *yfp-amp*. P1·IRE112 P_{ara} *yfp-amp* into XTL892

Table S2A. **DNA oligomers used to construct the *E. coli* strains in Table S1.**

Name	Sequence	Comments
XT833	TGTTCACAGGTTGCTCCGGGCTATGAAATAGAAAAATGAATCCGT TGAACTCCTAATTTTTGTTGACACTCTATC	make *attB<>tet-sacB*
XT834	CTTAAAGGTATTAAAAACAACTTTTTGTCTTTTTACCTTCCCGTTTC GCTATCAAAGGGAAAACTGTCCATATGC	make *attB<>tet-sacB*
XT835	AACAACTTTTTGTCTTTTTACCTTCCCGTTTCGCTTTCAACGGATTCA TTTTTCTATTTCATAGCCCGGA	make MG1655 *attB<>tet-sacB* clean deletion
XT836	CAACTTTTTGTCTTTTTACCTTCCCGTTTCGCTGCCTGCTTTTTTATA CTAACTTGTTCAACGGATTCATTTTTCTATTTCATAGCCCGG	invert the *attB*
XT841	ATCTCTTTAGCGCAGGAGCG	prophage single XTL886 check
XT842	CGCACATTGCCATGCTTGCG	prophage single XTL885 check
XT843	TTATTGCCATGCATCTGGTCAGTGCCCAAATGAGCGGAAATATGG AGGATTCCTAATTTTTGTTGACACTCTATC	make *bglG<>tet-sacB* (84.19')
XT844	AATTTTATTAATTGCAGCATTTCGCGCATTAACTGCGTGACACCTG CAACATCAAAGGGAAAACTGTCCATATGC	make *bglG<>tet-sacB* (84.19')
XT845	AGCATTTCGCGCATTAACTGCGTGACACCTGCAACCCTGCTTTTTTA TACTAACTTGATCCTCCATATTTCCGCTCATTTGGGCACTGAC	insert *bglG<>attB* toward ter (84.19')
XT846	AGCATTTCGCGCATTAACTGCGTGACACCTGCAACCAAGTTAGTAT AAAAAAGCAGGATCCTCCATATTTCCGCTCATTTGGGCACTGAC	insert *bglG*<> inverted *attB* toward oriC (84.19')
XT847	GAGGAAAATATTCCATTCTGGG	prophage single XTL875 check
XT848	GGTCATGTAACTCCTGGGGGC	prophage single XTL876 check
XT849	AGTTTAACAATATCAAGAAAGCACTAAGCGAAGCGAAATAACTAA ACCTTTCCTAATTTTTGTTGACACTCTATC	insert *tet-sacB* between *ydeN* and *ydeM* (34.05')
XT850	ACGTGcatCGCTATCTCGCTCAATAAGGCGGCGGAAAAATCCGCCG CATGATCAAAGGGAAAACTGTCCATATGC	insert *tet-sacB* between *ydeN* and *ydeM* (34.05')
XT851	AAAGCACTAAGCGAAGCGAAATAACTAAACCTTGCCTGCTTTTTTA TACTAACTTGCATGCGGCGGATTTTTCCGCCGCCTTATTGAGCG	insert *attB* between *ydeN* and *ydeM* toward ter(34.05')
XT853	AAAGCACTAAGCGAAGCGAAATAACTAAACCTTCAAGTTAGTATA AAAAAGCAGGCCATGCGGCGGATTTTTCCGCCGCCTTATTGAGCG	insert inverted *attB* between *ydeN* and *ydeM* (34.05')
XT855	GTCGCCCTCTGTTGCCCACC	prophage single XTL877 check
XT856	GTAGTAAAAACAGTGGACTGCCTT	prophage single XTL878 check
XT857	TCTTTTGCCACTACTAGCTTGACACCGCTTTTACCCTTCATTTGCAG CGCTCCTAATTTTTGTTGACACTCTATC	make *yfaH<>tet-sacB* (50.6')
XT858	CATTTTATCTGCTGGGCGCACTATTAGTGGTAGTGGCTGGTGGAA TCTATATCAAAGGGAAAACTGTCCATATGC	make *yfaH<>tet-sacB* (50.6')
XT859	CTTGACACCGCTTTTACCCTTCATTTGCAGCGCCAAGTTAGTATAAA AAAGCAGGATAGATTCCACCAGCCACTACCACTAATAGTGCGC	make *yfaH<>attB* toward ter (50.6')
XT860	CTTGACACCGCTTTTACCCTTCATTTGCAGCGCCCTGCTTTTTTATA CTAACTTGATAGATTCCACCAGCCACTACCACTAATAGTGCGC	make *yfaH*<> inverted *attB* toward ter (50.6')

Name	Sequence	Comments
Powell_1	GAGGTACCAGCGCGGTTTGATC	PCR to verify single copy of prophage integration
Powell_2	TTTAATATATTGATATTTATATCATTTTACGTTTCTCGTTC	PCR to verify single copy of prophage integration
Powell_3	ACTCGTCGCGAACCGCTTTC	PCR to verify single copy of prophage integration

Table S2B. **DNA oligomers for verifying single copy prophages.**

Final Strain	Strain	Location	PCR primers	Length (bp)	PCR primers	Length (bp)
XTL855	XTL848	17.4'	Powell_1 and Powell_2	221	Powell_2 and Powell_3	379
XTL856	XTL850	17.4'	Powell_1 and Powell_3	501	Powell_2 and Powell_3	379
XTL905	XTL875	34.05'	XT847 and Powell_3	689	Powell_2 and Powell_3	379
XTL906	XTL876	34.05'	XT848 and Powell_3	759	Powell_2 and Powell_3	379
XTL891	XTL877	50.6'	XT856 and Powell_3	581	Powell_2 and Powell_3	379
XTL892	XTL878	50.6'	XT855 and Powell_3	536	Powell_2 and Powell_3	379
XTL893	XTL885	84.19'	XT842 and Powell_3	556	Powell_2 and Powell_3	379
XTL894	XTL886	84.19'	XT841 and Powell_3	619	Powell_2 and Powell_3	379

Table S3. **Confirmation of the localization and orientations of prophage in different strains long-read sequencing.** Lysogenic strains bearing prophages in native and inverted orientations, at different sites on the chromosome, in the indicated backgrounds were sequenced (see Materials and Method). The bacterial genes adjacent to the prophage integration are indicated. BLAST nucleotide sequence identity between the lambda phage reference sequence and the prophages nucleotide sequence assemblies in the indicated strains is given.

**Strain**	**Background**	**Location of *attB* site**	**Orientation of prophage**	**Alignment length (bp)**	**Bacterial flanking genes**	**Sequence identity (%)**	**No. of mismatches**	**No. of gap openings**
XTL856	MG1655	17.4'	Native	48523	*ybhC, ybhB*	>99.9	30	4
XTL855	MG1655	17.4'	Inverted	48520	*ybhB, ybhC*	>99.9	29	4
XTL894	MG1655	84.19'	Native	48524	*bglG, bglF*	>99.9	31	4
XTL893	MG1655	84.19'	Inverted	48523	*bglF, bglG*	>99.9	31	4
XTL862	*recA-*	17.4'	Inverted	48524	*ybhB, ybhC*	>99.9	30	4
XTL863	*recA-*	17.4'	Native	48573	*ybhC, ybhB*	>99.9	90	74

Table S4. **Doubling times of lysogenic strains**. Doubling times of strains with prophages at native and inverted orientations, at different sites on the chromosome, under the experimental conditions (see Table S1).

Strain	Localization	Orientation	Medium	Doubling time (minutes)
XTL856	17.4’	Native	LB	22.2
XTL855	17.4’	Inverted	LB	22.7
XTL905	34.05’	Native	LB	22.6
XTL906	34.05’	Inverted	LB	23.3
XTL891	50.6’	Native	LB	22.7
XTL892	50.6’	Inverted	LB	22.9
XTL893	84.19’	Native	LB	25.2
XTL894	84.19’	Inverted	LB	23.0

Table S5. Sequences of smFISH probes. (Stellaris, with Cal590 tag).

Gene	*cI*	*cro*	*galk*
Probe sequences (5' to 3')	GGTTTCTTTTTTGTGCTCAT	ACAACCTCCTTAGTACAT	GTGTTTTTTCTTTCAGACTC
	CTCAAGCTGCTCTTGTGTTA	AGGGTTATGCGTTGTTCC	TAGCCAAATGCGTTGGCAAA
	AATTGCTTTAAGGCGACGTG	GCGCATTGCATAATCTTT	CTGAATGGTGTGAGTGGCAG
	GGGATAAGCCAAGTTCATTT	TAGCTGTCTTGGTTTGCC	CACCAATCAAATTCACGCGG
	ATCTTGTCTGCGACAGATTC	TGATATACGCCGAGATCT	ACGAAACCGTCGTTGTAGTC
	AATAAAGCACCAACGCCTGA	ATGGCCTTGTTGATCGCG	TGATAATCAATCGCGCAGGG
	GCATTTAATGCATTGATGCC	AATCTTTCGGCCTGCATG	GTGGTGCACAACTGATCACG
	TTTTTGCAAGCAATGCGGCG	CAGCGTTTATAGTTAAAA	ACGCGAACTTTACGGTCATC
	TCTTCAACGCTAACTTTGAG	TCCGCATAAACGCTTCCA	GCTGATTTTCATAATCGGCT
	TCTGGCGATTGAAGGGCTAA	CGGGAAGGGCTTTACCTC	GCATCGAGGGAAAACTCGTC
	GCTTCATACATCTCGTAGAT	CTGTTGTTTTTTTGTTAC	GTTTTCATGTGCGACAATGG
	AAGTGACGGCTGCATACTAA	AAGAGCGGGGTTATTTAT	CACGCCACGAACGTAGTTAG
	CAGGGTACTCATACTCACTT	TTTCAGGGCTGGAATGTG	TTACGCAGTTGCAGATGTTT
	CCTGCCTGAACATGAGAAAA	GGTTTAATTTGATGCCCT	TCCAGTGAAGCGGAAGAACT
	TCTAAGCTCAGGTGAGAACA	ATGCATACACCATAGGTG	TGCTGCAATACGGTTCCGAC
	CCGCATCACCTTTGGTAAAG	ATTGAATGTATGCAAATA	GTCCAGCGGCAGATGATAAA
	GAATCACTGGCTTTTTTGGT		TGACCGTTAAGCGCGATTTG
	TTCAACCTCAAGCCAGAATG		AGCCTACAAACTGGTTTTCT
	TTGGTGCGGTCATGGAATTA		GCGGAAATTAGCTGATCCAT
	CATTCCGTCAGGAAAGCTTG		AAGGCATGATCTTTCTTGCC
	TGCTCAGGGTCAACGAGAAT		CAGTGAGCGGCAATCGATCA
	GAAATCACCTGGCTCAACAG		TGGGCATGGAAACTGCTTTG
	TAAACTCATCACCCCCAAGT		GATGATGACGACAGCCACAC
	TCCCTGATCAGTTTCTTGAA		GGGTACGTTTGAAGTTACTG
	TTGTAAAAACACCTGACCGC		GTGTTGTATTCGCTGCCAAC
	ATTGGGTACTGTGGGTTTAG		GGTTTCGCACTGTTCACGAC
	ACAACTCTCATTGCATGGGA		TGGCTGCTGGAAGAAACGCG
	TGACTAGCGATAACTTTCCC		TTCAATGGTGACATCACGCA
	CAGCCAAACGTCTCTTCAGG		CATGCGCAACAGCGTTGAAC
			GGCGTTTTCAGTCAGTATAT
			ATACGTTTCAGGTCGCCTTG
			TGAGACTCCGCCATCAACTC
			GAAATCATCGCGCATAGAGG
			CAATTTGCGGCACGGTGATT
			TTGACGATTTCTACCAGAGT
			ACCTTTGTCGCCAATCACAG
			GGATCAGCGCGACGATACAG
			ATATTGTTCAGCGACAGCTT
			GTCTCTTTAATACCTGTTTT
			CTCCTTGTGATGGTTTACAA
